# Nano Modification of Antrodia Cinnamomea Exhibits Anti-Inflammatory Action and Improves the Migratory Potential of Myogenic Progenitors

**DOI:** 10.3390/cells11162512

**Published:** 2022-08-12

**Authors:** Mridula P. Menon, Yi-Hsuan Chien, Joy Thomas, Yu-Hsiang Yu, Chang-Tang Chang, Kuo-Feng Hua

**Affiliations:** 1 Department of Biotechnology and Animal Science, National Ilan University, Ilan 260007, Taiwan; 2 Department of Environmental Engineering, National Ilan University, Ilan 260007, Taiwan; 3 Department of Medical Research, China Medical University Hospital, China Medical University, Taichung 404327, Taiwan; 4 Department of Pathology, Tri-Service General Hospital, National Defense Medical Center, Taipei 11490, Taiwan

**Keywords:** muscle regeneration, Interleukin-6, long non-coding RNA, cell proliferation, cell migration, *Antrodia cinnamomea*, anti-inflammation

## Abstract

The skeletal muscle progenitors’ proliferation and migration are crucial stages of myogenesis. Identifying drug candidates that contribute to myogenesis can have a positive impact on atrophying muscle. The purpose of the study is to synthesize the *Antrodia cinnamomea* (AC)-β-cyclodextrin (BCD) inclusion complex (IC) and understand its in vitro pro-regenerative influence in murine skeletal C2C12 myoblasts. The IC was subjected to various nano-characterization studies. Fluorescent IC was synthesized to understand the cellular uptake of IC. Furthermore, 25 µg/mL, 12.5 µg/mL, and 6.25 µg/mL of IC were tested on murine C2C12 skeletal muscle cells for their anti-inflammatory, pro-migratory, and pro-proliferative action. The cellular internalization of IC occurred rapidly via pinocytosis. IC (252.6 ± 3.2 nm size and −37.24 ± 1.55 surface charge) exhibited anti-inflammatory action by suppressing the secretion of interleukin-6 and enhanced cell proliferation with promising cytocompatibility. A 12.5 μg/mL dose of IC promoted cell migration in 24 h, but the same dose of AC significantly reduced cell migration, suggesting modification by BCD. Molecular studies revealed that IC promoted C2C12 myoblasts migration by upregulating long non-coding RNA (lncRNA) NEAT-1, SYISL, and activating the pPKC/β-catenin pathway. Our study is the first report on the pro-proliferative and pro-migratory effects of BCD-modified extracts of AC.

## 1. Introduction

Antrodia cinnamomea (AC) is a well-known medicinal fungus indigenous to Taiwan. They are widely known for their anti-inflammatory, anti-oxidative, anti-cancer, hepatoprotective, pro-reproductive, and immunomodulatory action [1,2,3,4,5,6,7,8]. Due to their excellent medicinal properties, the extracts of AC are gaining crucial importance in the biomedical field. Recent studies on skeletal muscle cells show that extracts of AC protect against muscular atrophy and exhibit anti-fatigue effects [9,10]. The bioactive components present in the extracts of AC, such as sulphurenic acid, ergostatrien-3β-ol, dehydroeburicoic acid, and antcin K, have been reported to exhibit antihyperlipidemic and antidiabetic activity in skeletal muscle cells [11,12,13,14]. However, their influence on skeletal myogenesis has not been investigated so far.

The skeletal muscle plays a vital role in human health due to its irreplaceable role in metabolism and movement. The skeletal muscles are severely affected by various inflammatory diseases [15]. Inflammatory bowel disease (IBD) is a chronic inflammatory condition, and both forms of it, namely, ulcerative colitis (UC) and Crohn’s disease (CD), cause inflammation in the small and large intestine, respectively [16]. In IBD subjects, muscle wasting, sarcopenia (loss of muscle mass, strength, and function), and malnutrition have been reported [17]. The rate of prevalence of sarcopenia observed in UC and CD is 52% and 37%, respectively [18,19]. The level of circulating pro-inflammatory cytokines exhibits a systemic rise during IBD [16]. The inflammatory cytokines tumor necrosis factor-α (TNF-α) and interleukin (IL)-6 manifested in CD alter the body composition by negatively affecting protein metabolism [20]. They trigger the activation of the ubiquitin–proteasome pathway, apoptosis, and proteolytic muscle breakdown via the activation of the transcription factor, nuclear factor-kappa light chain enhancer of activated B cells (NF-κB) [20]. Therefore, for effective management of IBD, improving muscular health is an important consideration to upgrade the patient’s quality of life.

Several physiological cues trigger the multistep myogenesis characterized by progenitor cell proliferation, migration, fusion, and differentiation into mature myotubes [21,22]. Interestingly, the regulatory potential of numerous long non-coding RNAs (lncRNAs) in various stages of myogenesis at transcriptional and post-transcriptional levels has been identified recently [23]. The directional migration of muscle progenitor cells is critically important for myogenesis during muscle regeneration. Their movement to the injured site is necessary for muscle fiber repair [24]. Aberrancy in progenitor cell migration has severe implications for muscular diseases such as myopathies, progressive muscle wasting, and muscular dystrophies [24]. Therefore, therapeutic strategies to improve the migratory potential of skeletal muscle progenitor cells are critical.

Nanomaterials (1–100 nm in size) that act as transport modules for drugs in delivery applications are generally known as nanocarriers [25]. Cyclodextrins (CDs) are cyclic oligosaccharides naturally obtained from starch. They provide a suitable environment for the efficient encapsulation of various bioactive molecules and form nanoscale inclusion complexes [26]. The nanostructure of CDs is cylindrical with (1–4) glycosidic bonds linking the glucose residues. They have a hydrophilic exterior and hydrophobic interior, where non-polar drug molecules are efficiently encapsulated via hydrophobic interactions [26]. Although there are more than 1000 derivatives, the parent CDs (α, β, and γ) have gained widespread appreciation as carriers in drug delivery applications. β-cyclodextrin (BCD) with 7α-d-lucopyranose units has been reported to be biocompatible, non-cytotoxic, non-immunogenic, water-soluble, a high drug loading, and release action [27].

The purpose of this study is to modify AC using the nanocarrier BCD to unlock its full potential and try to make it suitable for skeletal muscle health improvement. The BCD-modified AC extracts were investigated for their influence on skeletal myogenesis in vitro using C2C12 myoblast cells. Since proliferation and migration are crucial stages of myogenesis, we were curious to understand whether nanomodified AC influenced these stages in any manner at the molecular level. We also synthesized fluorescent nanomodified AC to understand its cellular uptake. Our study is the first report to provide evidence on the anti-inflammatory, pro-proliferative, and pro-migratory action of BCD-complexed AC on C2C12 myoblast cells. Such evidence will amplify the usage of AC therapeutically with higher efficiency.

## 2. Materials and Methods

### 2.1. Cell Culture

Murine C2C12 myoblasts were obtained from American Type Culture Collection (ATCC Cat# CRL-1772, RRID:CVCL_0188). The cells were grown in a growth medium (Dulbecco’s modified Eagle’s medium (DMEM) with D-glucose, sodium pyruvate, and L-glutamine (GIBCO, Grand Island, NY, USA)) and supplemented with a 10% fetal bovine serum (FBS) (GIBCO, Grand Island, NY, USA) and 1% Antibiotic–Antimycotic (GIBCO, Grand Island, NY, USA) at 37 °C in a 5% CO_2_ incubator. The C2C12 myoblasts were differentiated into myotubes by switching them into a differentiating medium (DMEM with 2% Horse serum (GIBCO, Grand Island, NY, USA)) after the myoblasts reached 90% confluency. The successful formation of myotubes was observed 2–3 days post differentiation. All of the experiments using myotubes were performed on the 8th day post differentiation.

### 2.2. Synthesis of BCD-AC Conjugate (IC)

The AC fruiting body was obtained from REUI SEN Biotech CO., Ltd. (New Taipei City, Taiwan). Then, 50 g of the air-dried AC fruiting body were extracted with 1 L of 95% methanol for 3 days at room temperature, repeated five times. The extract was filtered and concentrated to give an ethanolic extract of AC. To synthesize the IC, BCD (Santa Cruz Biotechnology, Dallas, TX, USA) and the ethanolic extract of AC were dissolved in sterile water and absolute ethanol, respectively, at a 1:1 ratio. They were allowed to homogenate for 30 min at room temperature (RT) using an electromagnetic stirrer. After homogenizing, the dissolved solution of AC was added dropwise to the BCD solution under constant stirring at 100 RPM and allowed to homogenate overnight at RT. The solution was dried in the oven at 36 °C for 24 h [28,29]. The dried IC obtained was scraped using a sterile spatula and stored at 4 °C in a sterile Eppendorf. The total recovery percentage was calculated as per the following [30].

Total Recovery (%) = Recovered powder (mg)/Initial (beta cyclodextrin + AC) (mg) × 100

### 2.3. Synthesis of Fluorescent Nano-Drug Conjugate (AO-IC)

To develop the fluorescent nano-drug conjugate, acridine orange (AO) (Sigma Aldrich, St. Louis, MO, USA) and IC were dissolved together in a 2:1 ratio. Briefly, the accurately-weighed IC was dissolved in 20 mL of sterile double distilled water using a bath sonicator. It was then added dropwise to 50 µg/mL of a concentrated AO solution and homogenized for 48 h in the dark at RT over an electromagnetic stirrer at 110 RPM. The dark environment was created to avoid the loss of AO fluorescence. It was dried in an oven at 50 °C for 4 h to obtain the fluorescent AO-IC.

### 2.4. Characterization Studies on IC

To determine the water solubility and to compare them with the solubility profile of AC and BCD, different concentrations of IC (333 μg/mL, 430 μg/mL, 500 μg/mL, 600 μg/mL, 666 μg/mL, and 1 mg/mL) were dissolved in 3 mL of distilled water. The solution was subjected to bath sonication for 2 min at RT. The primary evaluation of the water solubility of IC was performed by visual observance [31,32]. To identify the charge, stability, size, and polydispersity index (PDI), zeta potential and dynamic light scattering (DLS) analysis was performed in a Zetasizer (Otsuka ELSZ-2000Z (Otsuka Electronics Co., Ltd., Japan) and Malvern Zetasizer (Malvern Instruments, Worcestershire, UK)). About 1 mg/mL of IC was dissolved in distilled water and was prepared to fit the instrument parameters. The morphological investigations on IC, AC, and BCD were carried out using a scanning electron microscope (SEM) (JSM-IT100, Joel, Tokyo, Japan). Fourier transform infrared spectroscopy (FT-IR) analysis was performed using a Nicollet iS 10 FTIR spectrometer (Thermo Fisher Scientific Inc., Madison, WI, USA) within the range of 4000–400 cm^−1^. To understand the temperature stability, thermogravimetric (TGA)/differential scanning calorimetric (DCS) analysis was performed using an STA 6000 Simultaneous Thermal Analyzer (Perkin Elmer, Waltham, MA, USA). To obtain the fluorescent measurements, 200 µL of the samples (200 µg/mL) were carefully added to a 96-well plate, and the relative fluorescence was measured using the Spectra Max M2 (Molecular Devices, Sunnyvale, CA, USA) with SoftMax pro-5.4.1. software (Molecular Devices, Sunnyvale, CA, USA). The excitation and emission wavelength were fixed at 320 nm and 360–500 nm, respectively. To understand the absorption characteristics, UV-Visible spectroscopic analysis was performed on the aqueous sample solutions (200 µg/mL) using the UV-3900/3900H Spectrophotometer (Hitachi Ltd., Tokyo, Japan) in the range of 250–600 nm. The in vitro dissolution studies of AC and IC were performed according to the procedure described by Wang et al. [29]. Briefly, 10 mg of AC or IC was added to 50 mL of the dissolution medium (phosphate-buffered saline (PBS) pH 7.4) placed at RT and allowed to stir continuously at 100 RPM. At the set period, 3 mL of the sample was withdrawn and measured spectrometrically using a UV-visible spectrophotometer at 264 nm. An equal volume of fresh PBS pH 7.4 was replaced after each sample estimation. The study was performed in triplicate, and the data show the average of three independent experiments. From the analysis, the cumulative drug release is calculated based on the following formulas [29,30,31]:

Concentration of drug (µg/mL) (y) = (slope (m) × absorbance (x)) ± intercept (c).

The amount of drug released (mg/mL) can be calculated as follows:

Amount of drug released = Concentration × Dissolution medium volume/1000.

The percentage of drug release is calculated as follows:

Drug release (%) = Amount of drug released (mg)/Drug dose (mg) × 100.

The cumulative drug release percentage is calculated as follows:

Cumulative drug release (%) = Volume of sample withdrawn (mL)/Dissolution.

medium volume × p(t − 1) + pt

where pt is the percentage release at time point ‘t’, and P(t − 1) is the percentage release at the time before ‘t’.

### 2.5. Bioassays

#### 2.5.1. Cell Viability Assay

The cytotoxicity levels of IC and BCD were determined on C2C12 myoblasts using a microculture tetrazolium (MTT) assay. After culturing the cells in a 96-well plate for 24 h, different concentrations (200 µg/mL, 100 µg/mL, 50 µg/mL, 25 µg/mL, 12.5 µg/mL, and 6.25 µg/mL) of IC or BCD were added to the test cells and incubated for 24 h. Later, the cells were allowed to react with 10 µL of 5 mg/mL MTT (Calbiochem, Darmstadt, Germany) for 4 h. The medium was discarded at the end of incubation, and 100 µL of the destaining solution, DMSO, was then added. After 10 min of incubation at room temperature, the absorbance was measured using an absorbance microplate reader (Bio Rad Laboratories Inc., Hercules, CA, USA) at 570 nm. The percentage cell viability was calculated as follows:

Cell viability (%) = (O.D. of test sample − O.D. of blank)/O.D. of solvent ∗ 100

#### 2.5.2. Proliferation Assay

The C2C12 myoblasts (4 × 105/mL) were incubated with 200 µg/mL, 100 µg/mL, 50 µg/mL, 25 µg/mL, 12.5 µg/mL, and 6.25 µg/mL of IC for 24 h. The untreated cells were monitored as the control. The cell numbers before and after the treatment were counted using the Trypan blue exclusion method.

#### 2.5.3. Anti-Inflammatory Studies

##### Protein Denaturation Inhibition Study

To understand the protein denaturation inhibition potential of the synthesized IC, 450 μL of 5% aqueous solution of bovine serum albumin (BSA) was added to 50 μL of different concentrations of IC (6.25 μg/μL, 12.5 μg/μL, and 25 μg/μL) and mixed well. The reaction mixtures were later incubated at 37 °C for 20 min in a water bath, followed by 3 min of heating at 57 °C. The reaction mixtures were cooled to RT before adding 2.5 mL of 1× PBS (pH 6.3). 5-amino salicylic acid (5 ASA) and 1× PBS (pH 6.3) were used as the positive and negative control, respectively. The absorbance of the solution was analyzed using a Spectra Max M2 (Molecular Devices, Sunnyvale, CA, USA) spectrophotometer at 660 nm. The percentage inhibition of protein denaturation was calculated as follows [33,34]:

Percent inhibition = 100 × (1 − O.D of test/O.D. of control)

##### Enzyme-Linked Immunosorbent Assay (ELISA)

The C2C12 myoblast cells were initially tested for their ability to produce pro-inflammatory cytokines (TNF-α, IL-6, nitric oxide, and IL-1β) by stimulating the cells with 100 ng/mL of lipopolysaccharide (LPS) (from Escherichia coli O111:B4 strain (L2630)) for 24 h. To observe IL-1β production, the LPS-stimulated cells were further activated by ATP (5 mM) for 30 min. The concentration of pro-inflammatory cytokines from the cell supernatant was assayed by a commercially available ELISA kit (Invitrogen mouse ELISA Kit, Life Technologies Corp., Carlsbad, CA, USA) according to the manufacturer’s instructions. The level of NO in the culture supernatant was determined using the Griess reaction. The procedure for Griess reaction has been described in detail in our previous paper by Hua et al. [35]. To study the ability of IC and AC to suppress IL-6 production, the C2C12 myoblasts and myotubes (8th-day post differentiation) were cultured in 6-well plates for 24 h and treated with different concentrations (25 µg/mL, 12.5 µg/mL, and 6.25 µg/mL) of IC and AC for 30 min and 3 h. Later, they were stimulated with 100 ng/mL of LPS for 24 h. The concentration of IL-6 from the cell supernatant was assayed by a commercially available ELISA kit (Invitrogen mouse-IL-6 ELISA Kit, Carlsbad, CA, USA) according to the manufacturer’s instructions.

#### 2.5.4. In Vitro Migration and Wound Healing Assay

The C2C12 myoblasts (1 × 105/mL) were cultured in 6-well plates in a growth medium with 10% FBS. After reaching the required confluency, the medium was replaced with a low serum medium (2% FBS) to encourage migration and avoid the proliferation of the cells. To this, varying doses (25 µg/mL, 12.5 µg/mL, and 6.25 µg/mL)) of IC or AC were added. Later, using a sterilized 200 µL pipette tip, a straight-line wound was inflicted in the cell monolayer. The wound closure space was measured at different time intervals (0, 6, 12, 18, and 24 h) using an Olympus CK30 phase-contrast inverted microscope (USA). The images were analyzed using NIH ImageJ software (RRID:SCR_003070). The migration parentage was calculated as follows [30]:

Cell migration (%) = Distance at time ‘t’/Initial Distance (t = 0) ∗ 100

#### 2.5.5. Western Blot

The C2C12 myoblasts were treated with different concentrations (25 µg/mL and 12.5 µg/mL) of IC or AC for 30 min before stimulating them with 100 ng/mL of LPS for 24 h. The whole-cell lysates were prepared using NP-40 lysis buffer (1 µg/mL of leupeptin, 1 mM of PMSF, 2.5 mM of sodium pyrophosphate, 1 mM of Na_3_VO_4_, 1 mM of β-glycerol phosphate, 1% sodium deoxycholate, 1% NP-40, 1 mM of EGTA/EDTA, 20 mM of Tris-HCl (pH 7.4), and 150 mM of NaCl) and the protein concentrations in each sample were determined using a Bradford assay. The antibodies against N-cadherin (Abcam Cat# 2447-1, RRID:AB_1267002), p-PKC (Santa Cruz Biotechnology Cat# sc-136018, RRID:AB_2168554), actin (BD Biosciences Cat# 612656, RRID:AB_2289199), and β-catenin (Cell Signaling Technology, Danvers, MA, USA. Cat# 19807, RRID:AB_2650576) were used at 1:1000 dilutions. The procedure followed for Western blot has been described in detail in our previous paper by Chao et al. [30].

#### 2.5.6. Nanodrug Uptake Study

##### AO-IC Optimization and Time-Course Analysis

To optimize the AO-IC required for the efficient uptake by the myoblast cells, 4 × 105 cells were cultured in 6-cm dishes with DMEM supplemented with 10% FBS. Upon reaching the required confluency, different concentrations of AO-IC were added and incubated for 3 h. Further, the culture dishes were transferred for imaging using a fluorescence microscope (Zeiss Axioimager.D2, Carl Zeiss, GmbH, Oberkochen, Germany) after washing twice with PBS. The images were obtained using a digital camera (HyperS300, Carl Zeiss, GmbH). To understand the efficiency of the fluorescent complexation with IC, 400,000 cells were cultured in a 6-cm dish with 2 mL of medium. To the confluent cells, 5 µL of AO-IC, IC (25 µg/mL), and AO (25 µg/mL) was added and incubated for 3 h. The culture dishes were further processed for imaging. The cellular uptake of IC as a function of time was determined by incubating the C2C12 myoblasts with 5 µL of AO-IC for different time intervals (5, 30, 120, and 180 min). The cells were washed with PBS and proceeded for fluorescent imaging. The image was examined using NIH Image J software. The corrected total cell fluorescent (CTCF) values were obtained using the following formula.

CTCF = Integrated density − Area × Background fluorescence

The uptake of IC as a function of time was further confirmed by FACS analysis. The cells treated with 5 µL of AO-IC at different time intervals (5, 30, 120, and 180 min) were washed twice with ice-cold PBS (1×) solution and trypsinised for 2 min. The cells were then harvested by centrifugation at 950 RPM for 5 min and redispersed in 1 mL of PBS. The samples were analyzed by a BD Accuri C6 C sampler flow cytometer and measured with the FL3-A channel.

##### Nanodrug Internalization Analysis

The mechanism involved in the cellular uptake of IC was studied by using specific endocytosis inhibitors. The C2C12 myoblasts were incubated in the presence of 25 µg/mL of colchicine (Merck, Darmstadt, Germany) and 0.5 M sucrose (Sigma Aldrich, St. Louis, MO, USA) for 30 min. After washing the cells twice with 1× PBS, the cells were incubated with IC (25 µg/mL) for 2 h. The cells were then monitored by FACS and fluorescent imaging. The image analysis was performed using NIH Image J software (NIH, Bethesda, MD, USA) and the CTCF values were calculated as described in Section 2.5.6.

#### 2.5.7. RT-PCR Studies

For mRNA expression studies, the confluent C2C12 myoblasts were treated with 25 µg/mL of IC for 30 min, followed by LPS stimulation (100 ng/mL) for 24 h. The cells with/without LPS stimulation were observed as the positive/negative control. The total RNA from the cells was extracted using Trizol reagent (Ambion, Life technologies, Carlsbad, CA, USA). The extracted RNA was reverse transcribed into cDNA using a Revert Aid first-strand cDNA synthesis kit (Thermo Scientific, Waltham, MA, USA). Using SYBR Green master mix (Vazyme biotech Co. Ltd., Nanjing, China), the expression of mRNA was studied by quantitative real-time PCR using the StepOne Real-time PCR system (Applied Biosystems, Foster city, CA, USA). The primer sequences used in the study are the following: lncRNA SYISL F: CTCGTGGTCCCTCCCTGTAA, R: GTCTGCGTGCTCCTGTGGTT. lncRNA NEAT1 F: GGGAAGGGTGACATTGAAAA, R: CTCCCCAGCTTCACTTCTTG and lncRNA MEG3 F: TTGAGTAGAGACCCGCCCTC, R: CTGTGCTTTGGAACCGCATC. GAPDH was employed as an internal control in the study. The relative gene expression of the gene was calculated by the Ct (2−ΔΔCt) method.

### 2.6. Statistical Analysis

All of the data presented in the study are expressed as the mean ± SEM of three independent experiments (*n* = 3). SAS software was used for the statistical analysis of the data in the present study. The student *t*-test (two-tailed) and one-way ANOVA (Dunnett’s multiple comparison test) were used to analyze the statistical significance of the data. The probability values (*p* < 0.05) were considered statistically significant in all of the cases.

## 3. Results

### 3.1. Synthesis of BCD-AC Inclusion Complex (IC)

**Figure 1:**
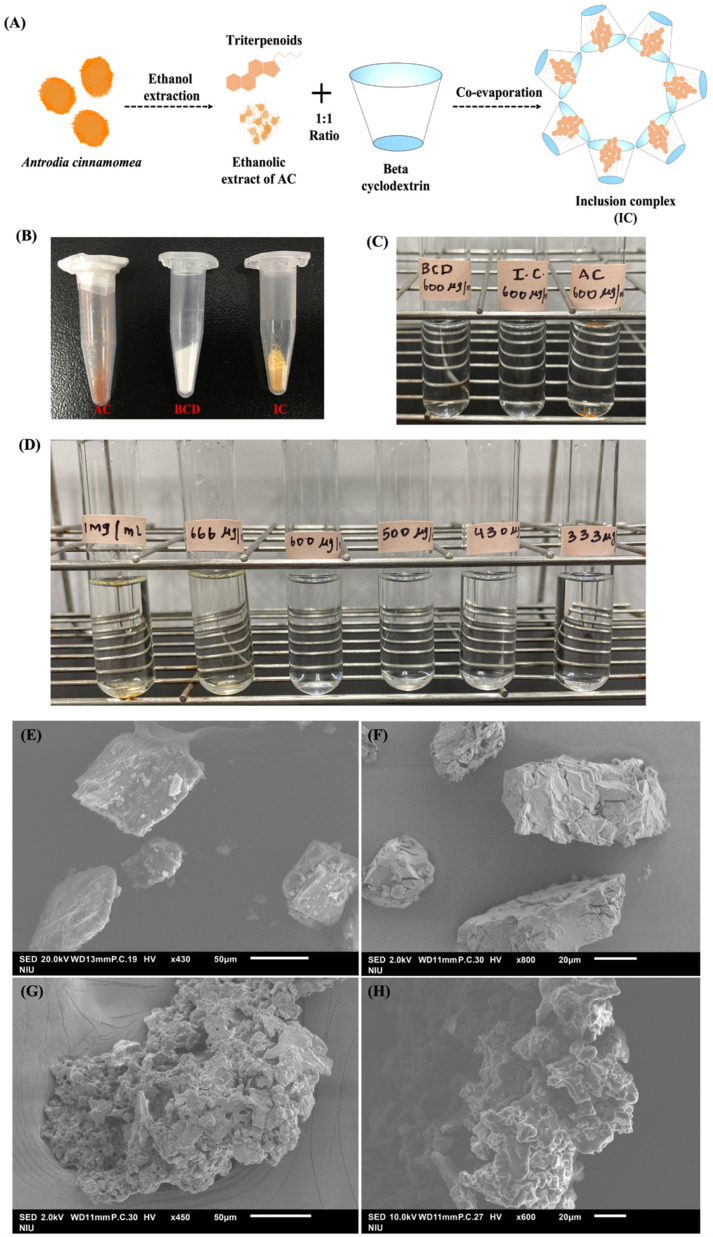
(**A**) Schematic diagram showing the synthesis of IC (**B**) Co-evaporation of ethanolic extract of AC (brown) and BCD (white) resulted in the formation of the IC (yellow). (**C**) Water solubility analysis of BCD, AC, and IC. (**D**) Water solubility analysis of different concentrations of IC. Morphological analysis of (**E**) BCD, (**F**) AC (**G**), and (**H**) IC using SEM.

To transform the hydrophobic nature of the AC extract, we attempted to complex it with BCD using the co-evaporation technique. This resulted in the formation of a molecular inclusion complex (IC) between BCD and AC (Figure 1A,B). The visual analysis of the water solubility of IC indicated that the lower concentrations of IC (333 μg/mL, 430 μg/mL, 500 μg/mL, and 600 μg/mL) exhibited complete water solubility compared to the higher concentrations (666 μg/mL and 1 mg/mL) (Figure 1D). The maximum water solubility of IC was observed at a concentration of 600 μg/mL. The analysis of the water solubility of the parent compounds of IC indicated that 600 μg/mL of BCD displayed high water solubility, whereas AC (600 μg/mL) exhibited water insolubility (Figure 1C). Thus, the hydrophobic nature of AC was transformed into a hydrophilic nature upon their interaction with BCD due to inclusion complex formation. The prepared formulation exhibited a recovery percentage of 73.3 ± 2.8.

### 3.2. Nano Characterization Studies on Prepared IC

**Figure 2:**
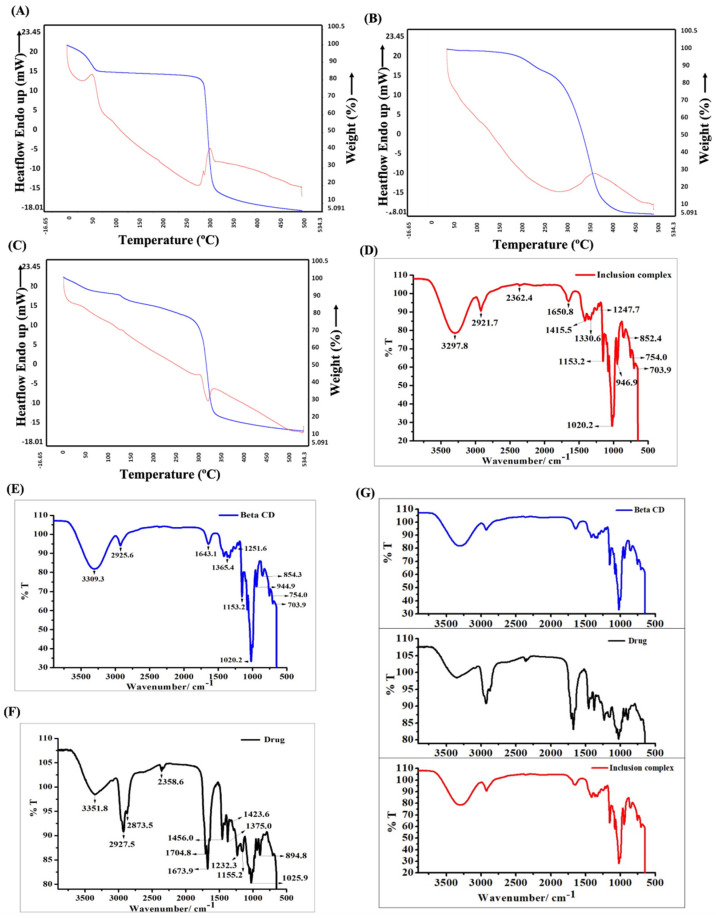
TGA (Blue)/DSC (Red) analysis of (**A**) BCD, (**B**) AC, and (**C**) IC. FT-IR spectral analysis of (**D**) IC, (**E**) BCD, and (**F**) AC. (**G**) Merged FT-IR spectra of BCD, AC (drug), and IC.

**Figure 3:**
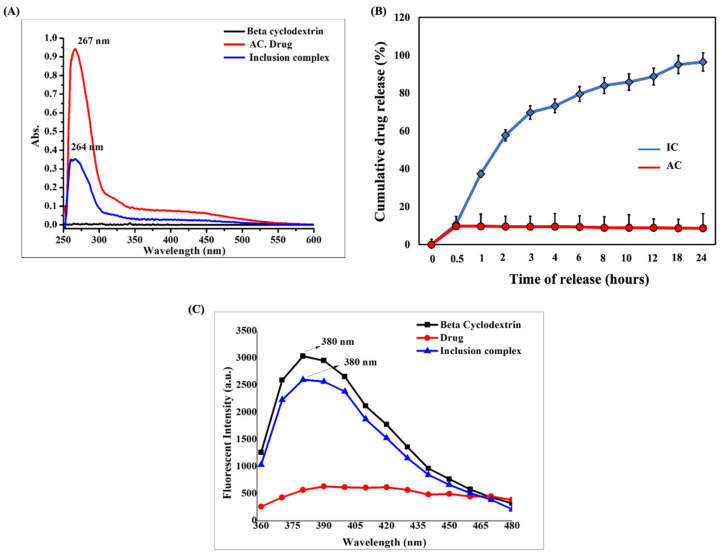
(**A**) UV-visible absorption spectra of BCD, AC, and IC. (**B**) In vitro dissolution profile of AC and IC in PBS (pH 7.4) (**C**) Fluorescent spectra of BCD, AC, and IC.

The zeta potential and DLS analysis indicated that IC exhibited a hydrodynamic size of 252.6 ± 3.2 nm with a dispersity index of 0.340 ± 0.05 and a strongly negative surface charge of −37.24 ± 1.55 (Figure S1a,b). According to the SEM analysis, IC appeared to be porous and amorphous in nature, with spherical beads on the surface (Figure 1E–H). A disappearance or shift in the melting point of the drug upon complexation with BCD indicates successful inclusion complex formation [36]. The compounds with higher melting points exhibited lower solubility profiles. In our study, we observed that the intermolecular interaction between AC and BCD led to the improved solubility of the resulting inclusion complex. This is also supported by the TGA analysis, where the complexation of AC with BCD shifted the melting point of AC from 370 °C to 340 °C. The weight loss pattern observed in IC was similar to that of BCD. The weight loss of the compound refers to its melting point. The weight loss occurs when there is a decomposition of the material, and the decomposition of the material initiates at its melting point. A >98% weight loss was observed in AC and BCD upon heating to 500 °C, whereas only a 90% weight loss was observed in IC at 500 °C. The differences in the endothermic peaks observed between AC, BCD, and IC in the DSC analysis also indicate successful IC formation (Figure 2A–C). The broad endothermic peak observed in AC and BCD at 260 °C has been shifted to a sharper endothermic peak at 330 °C upon the inclusion complex formation. The FTIR analysis showed that the IR spectrum of IC was similar to that of BCD and was not identical to AC (Figure 2G). The prominent peaks of AC appeared to be shifted to lower and higher frequencies with reduced intensities. The disappearance of the characteristic peaks of AC at 2358.6 cm^−1^, 1673.9 cm^−1^, 1456.0 cm^−1^, 1025.9 cm^−1^, and 894.8 cm^−1^ in IC indicated a successful inclusion complex formation with BCD (Figure 2D–F). The UV-visible absorption studies indicated that IC depicted a strong absorption maximum at 264 nm, which is profoundly different from that of AC. The absorption difference observed in AC and IC shows the successful inclusion of AC in BCD (Figure 3A). The in vitro dissolution studies confirmed that the association of AC with BCD enhanced the dissolution profile of IC, and complete drug release was observed within 24 h (Figure 3B). The hydrophobic nature of AC made it completely insoluble in the dissolution medium. The higher solubility of IC in the medium could also be due to its amorphous nature upon interaction with BCD. The fluorescent spectroscopic analysis indicated that the association of BCD with AC enhanced the fluorescent intensity of IC compared to AC (Figure 3C). From these studies, we confirmed the successful inclusion complex formation between BCD and AC. Detailed information on nano-characterization studies is provided in the supplementary information Section S1.

### 3.3. IC Exhibit Cytocompatibility and Enhance Proliferation of C2C12 Myoblasts

**Figure 4:**
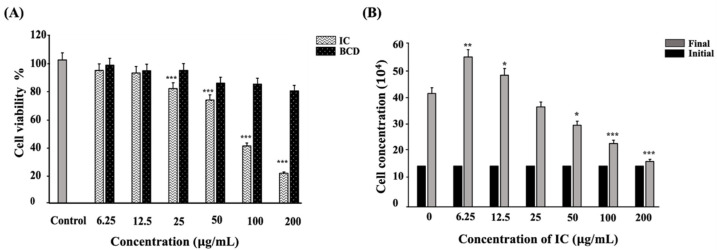
Lower concentrations of IC exhibited cytocompatibility and improved proliferation (**A**) C2C12 myoblasts treated with BCD and IC separately for 24 h prior to MTT exposure and measured spectrophotometrically at 570 nm. (**B**) C2C12 myoblasts were treated with IC, incubated for 24 h, and counted using trypan blue exclusion method to assess the cell proliferation. All the experiments were repeated thrice, and data were shown as mean ± SD; n = 3. *, ** and *** shows a significant difference at *p* < 0.05, *p* < 0.01, and *p* < 0.001, respectively, compared to the control (Two-tailed *t*-test).

We observed that lower concentrations of IC (6.25 µg/mL, 12.5 µg/mL, and 25 µg/mL) did not negatively affect the cell viability compared to the higher concentrations (50 µg/mL, 100 µg/mL, and 200 µg/mL) (Figure 4A). Hence, for further studies, lower doses of IC were considered suitable. All of the concentrations of BCD exhibited more than 80% cell viability, indicating their suitability in the formulation (Figure 4A). The proliferation assay indicated that lower doses of IC (6.25 µg/mL and 12.5 µg/mL) enhanced the proliferation of cells compared to the untreated control cells (Figure 4B). Therefore, we concluded that the BCD-included formulation is suitable for in vitro studies on C2C12 myoblasts and that lower doses of IC are cytocompatible and pro-proliferative.

### 3.4. Cellular Internalization of IC Involves Pinocytosis Pathway

**Figure 5:**
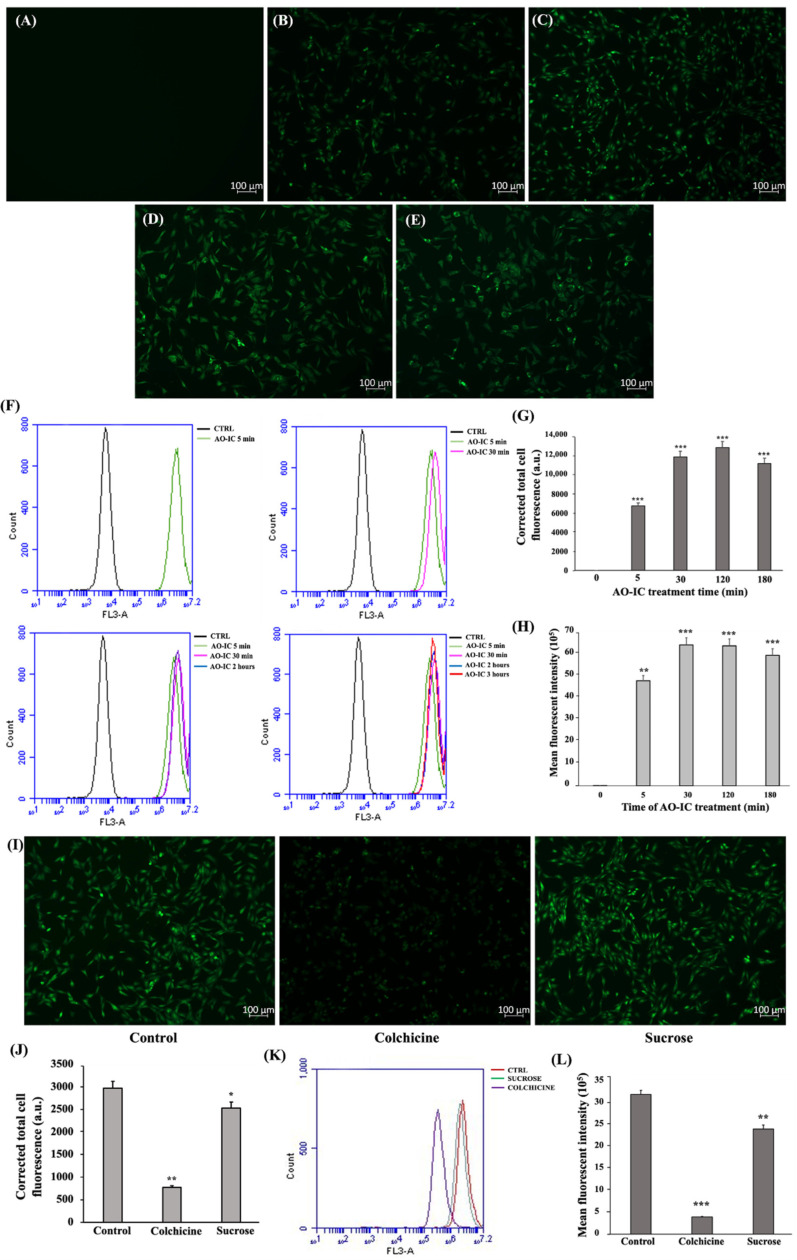
C2C12 myoblasts internalize IC via the pinocytosis pathway. Cell internalization of AO-IC as a function of time analyzed by fluorescence microscopy. (**A**) 0 min, (**B**) 5 min, (**C**) 30 min, (**D**) 120 min, and (**E**) 180 min. (**F**) FACS analysis on cellular uptake of AO-IC. (**G**) CTCF measured upon the cell internalization of AO-IC with respect to time and (**H**) mean fluorescent intensity measured. (**I**) Fluorescent microscopic images of C2C12 myoblast cells treated with or without colchicine and hyperosmotic sucrose, followed by AO-IC incubation for 2 h. (**J**) CTCF measured upon AO-IC internalization after endocytosis inhibitor treatment. (**K**) FACS analysis and (**L**) mean fluorescent intensity measurement post cellular internalization of AO-IC after endocytosis inhibitor treatment. The study was repeated three times, and the results were shown as mean ± SD; n = 3. *, ** and *** show a significant difference at *p* < 0.05, *p* < 0.01, and *p* < 0.001, respectively, compared to the control (Two-tailed *t*-test).

The fluorescent property of the formulated AO-IC was investigated using fluorescent spectrophotometer and fluorescent microscopy (Figure S2a–e). The time course analysis indicated that the internalization of AO-IC initiated within 5 min (Figure 5B) and increased with the progression of time (Figure 5C–E). The control without the AO-IC treatment is shown in Figure 5A. The internalization of AO-IC was confirmed by the FACS analysis (Figure 5F). These results suggested that the cellular internalization of the formulated AO-IC was highly effective and rapid. The AO-IC uptake pathway investigated by fluorescent microscope and FACS analysis is shown in Figure 5I–L. We noted that the hyperosmotic conditions created by excess sucrose inhibited IC uptake slightly (1.2 times). Interestingly, the colchicine treatment significantly reduced the IC uptake (six times) compared to the control cells (Figure 5J). The FACS analysis confirmed the contribution of the pinocytosis pathway in the cellular internalization of IC (Figure 5K–L). Therefore, we concluded that the complete cellular internalization of IC is mediated by the energy-dependent endocytosis process.

### 3.5. IC Suppressed the Expression of the Pro-Inflammatory Cytokine, IL-6 in Myoblasts

**Figure 6:**
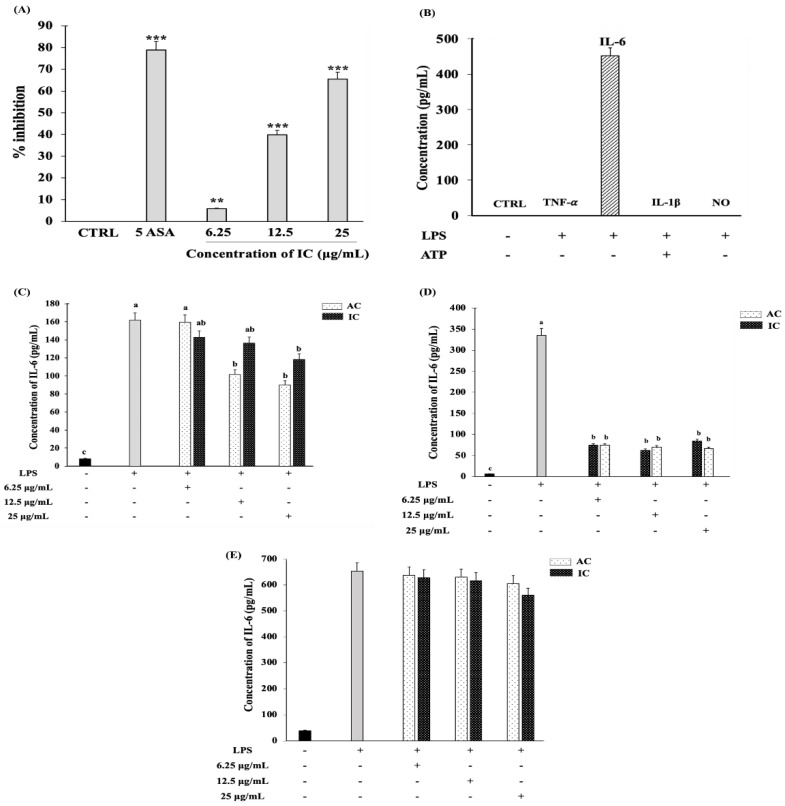
IC exhibited anti-inflammatory activity and suppressed the IL-6 production in C2C12 myoblasts cells. (**A**) Ability of different concentrations of IC to inhibit protein denaturation. (**B**) Pro-inflammatory cytokines production in C2C12 myoblast cells stimulated with 100 ng/mL LPS for 24 h. For the production of IL-1β, the LPS-stimulated cells were further activated with ATP. Cells were treated with 25 µg/mL, 12.5 µg/mL and 6.25 µg/mL concentrations of IC and AC for (**C**) 30 min and (**D**) 3 h before stimulating with 100 ng/mL LPS for 24 h. (**E**) IL-6 suppressive effect of IC and AC in C2C12 myotubes. After reaching the required confluency, the C2C12 myoblasts were switched to 2% HS medium to initiate the differentiation. After 8 days of differentiation, the C2C12 myotubes were subjected to IC and AC treatment for 3 h prior to 100 ng/mL LPS stimulation for 24 h. The IL-6 levels in culture supernatant were measured by ELISA. The study was repeated three times, and the results were shown as mean ± SD; *n* = 3. ** and *** shows a significant difference at *p* < 0.05, *p* < 0.01 and *p* < 0.001, respectively, compared to the control (Two-tailed *t*-test). The statistical comparison between groups was performed using One-way ANOVA (Dunnett’s multiple comparisons test). abc indicate significant differences between the groups. Statistically different groups are denoted by different letters and different groups of letters. Similar letters and similar groups of letters denote no statistical difference between them.

The denaturation of proteins occurs commonly during inflammatory conditions. The major mechanism of action reported in several anti-inflammatory drugs is the protection of the proteins from denaturation. Understanding the protein denaturation inhibition potential of a candidate drug is critically important for understanding its anti-inflammatory ability [33,34]. The protein denaturation study indicated that IC exhibited concentration-dependent inhibition of protein denaturation (Figure 6A). The commercially available anti-inflammatory drug, 5 ASA, exhibited a maximum inhibition of protein denaturation. The LPS stimulation of C2C12 myoblast cells did not enhance the production of TNF-α, nitric oxide, and IL-1β in the culture supernatant. However, the 24 h stimulation of C2C12 myoblast cells with LPS caused a significant production of IL-6 in the supernatant (Figure 6B). Therefore, the ability of IC to suppress the production of IL-6 was studied further to understand their anti-inflammatory activity. To gain insight into the anti-inflammatory action of the developed IC, ELISA was performed. The LPS stimulation of C2C12 myoblasts induced the secretion of IL-6, and this effect was reduced by the IC 0.5 h pre-treatment in a dose-dependent manner. A similar trend in IL-6 reduction was observed in the case of AC (Figure 6C). Interestingly, prolonging the drug exposure on myoblasts to 3 h prior to LPS stimulation significantly reduced the IL-6 expression levels in the case of both IC and AC (Figure 6D). In addition, 24 h LPS stimulation of C2C12 myotubes induced the secretion of IL-6, and this effect was reduced by IC 3 h pre-treatment in a dose-dependent manner (Figure 6E). Hence, we concluded that IC exhibited profound anti-inflammatory activity by significantly inhibiting the IL-6 secretion in C2C12 myoblasts. The complexation of BCD with AC maintained their anti-inflammatory activity and slightly enhanced their potential to inhibit IL-6 in C2C12 myotubes.

### 3.6. IC Enhanced Myoblasts Migration by Influencing the lncRNA Expression

**Figure 7:**
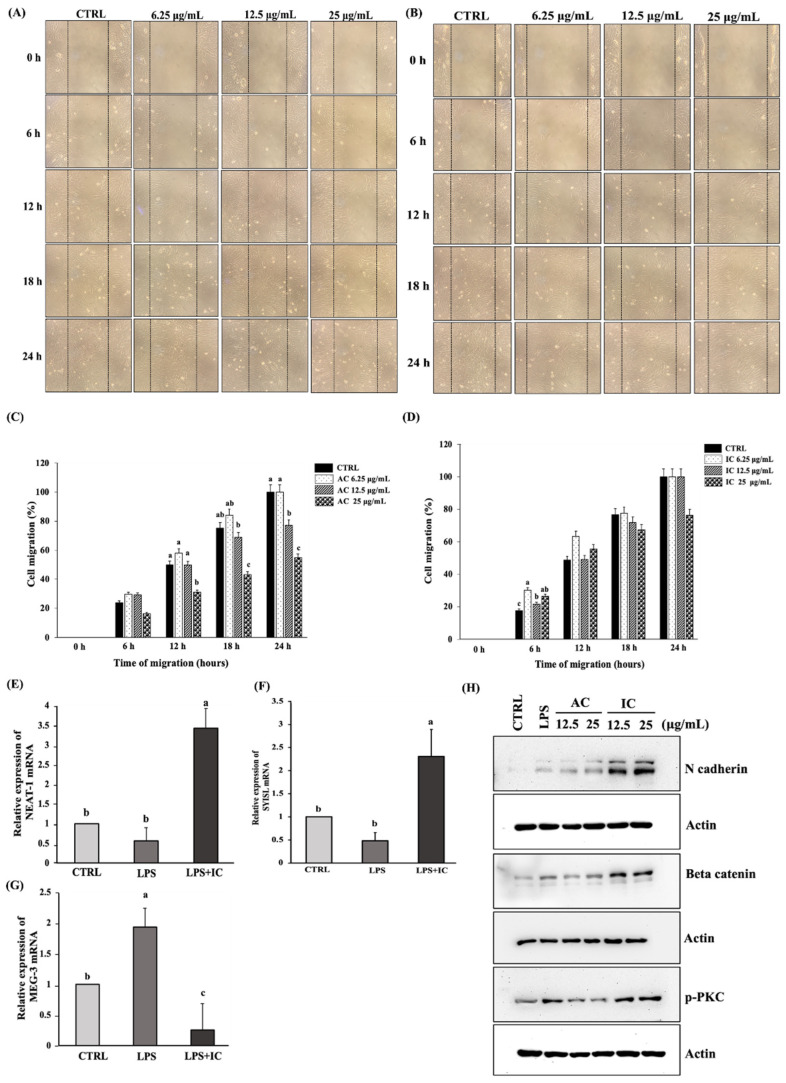
IC influences the lncRNAs expression to promote the migration and proliferation of C2C12 myoblast cells. Wound healing assay performed on myoblasts treated with 6.25 µg/mL, 12.5 µg/mL, and 25 µg/mL of (**A**) AC and (**B**) IC with respect to time (0 h, 6 h, 12 h, 18 h, and 24 h) visualized using a microscope. The percentage cell migration calculated on myoblasts treated with (**C**) AC and (**D**) IC with respect to time. The influence of IC on the lncRNA expression in C2C12 myoblasts was analyzed using RT-PCR study. The cells were treated with 25 µg/mL of IC followed by LPS treatment, RNA extraction, and cDNA synthesis. The RT-PCR analysis was carried out to determine the effect of IC on (**E**) lncRNA NEAT-1, (**F**) lncRNA SYISL, and (**G**) lncRNA MEG-3 expression. (**H**) The effect of IC on the expression of migratory proteins was studied by western blot analysis. The study was repeated three times, and the results were shown as mean ± SD; *n* = 3. The statistical comparison between groups was performed using One-way ANOVA (Dunnett’s multiple comparisons test).abc indicate significant differences between the groups. Statistically different groups are denoted by different letters and different groups of letters. Similar letters and similar groups of letters denote no statistical difference between them.

To investigate the effect of IC on cell migration, an in vitro wound healing assay was performed. AC at 12.5 µg/mL reduced myoblasts migration at 18 and 24 h, while AC at 25 µg/mL reduced myoblasts migration at 12, 18, and 24 h (Figure 7A,C). Interestingly, the BCD complexation of AC enhanced the migration potential of C2C12 myoblasts cells (Figure S3a,b). The myoblast migration was only significantly reduced by 25 µg/mL IC at 24 h (Figure 7B,D). These results indicated that IC has a less inhibitory effect on myoblasts migration than AC.

**Figure 8:**
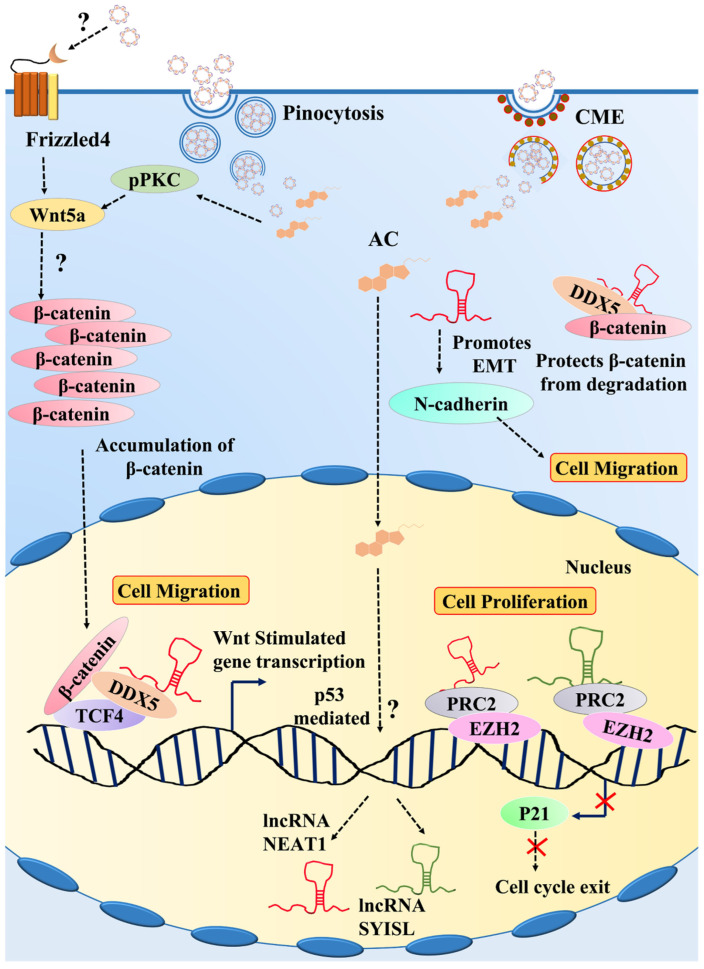
Demonstration of the signaling pathway triggered by IC in C2C12 myoblasts.

In addition, we studied the expressions of major lncRNAs involved in the migration of C2C12 myoblasts. We observed that the LPS stimulation negatively regulated the expression of lncRNA nuclear-enriched abundant transcript 1 (NEAT-1) (Figure 7E) and SYNPO2 intron sense overlapping lncRNA (SYISL). (Figure 7F) whereas it enhanced lncRNA maternally enriched transcript 3 (MEG3) expression (Figure 7G). Intriguingly, we noted that IC reversed the inhibitory effect of LPS on LncRNAs NEAT 1 and SYISL and considerably suppressed the lncRNA MEG3 expression (Figure 7E–G). Since lncRNA NEAT 1 and SYISL are predominantly involved in promoting the myoblast migration and proliferation than lncRNA MEG3, we concluded that IC promoted C2C12 myoblast migration by enhancing the expression of lncRNA NEAT 1 and SYISL. Further, we attempted to investigate whether IC influenced the expression of the major migratory proteins involved in the signaling pathways of lncRNA NEAT 1 and SYISL. We observed that IC potentially enhanced the protein expression of N-cadherin, β-catenin, and p-PKC (Figure 7H). Therefore, we concluded that IC enhanced the migration of C2C12 myoblast cells by activating the lncRNAs responsible for promoting β-catenin/pPKC activity. Figure 8 shows the pathway influenced by IC in C2C12 myoblast cells that ultimately resulted in their migration and proliferation.

## 4. Discussion

BCD and its derivatives have been successfully used as carriers of numerous water-insoluble drugs to improve their solubility and bioavailability [30,36,37,38,39,40,41,42]. In our study, we showed that the AC/BCD inclusion complex could improve the solubility and bioavailability and influence the signaling pathways at molecular levels in murine C2C12 skeletal myoblasts. The apolar nature of the BCD cavity energetically favors the water molecules present in them. This, in turn, favors their replacement with the hydrophobic AC in the cavity through intermolecular forces and non-covalent bonds [43]. The BCD enhances the solubility of the incorporated hydrophobic AC in the dissolution medium in multiple ways. It acts as a surfactant, enhances the wetting of the hydrophobic surface of AC, and reduces the interfacial tension between the solid fractions of AC and the dissolution medium, thereby ensuring the increased solubility of the drug [44].

The cell entry of drug carriers is a crucial process influenced by the size, surface charge, shape, and rigidity of the carriers [45]. Colchicine inhibits pinocytosis by preventing the vesicular formation and microtubule trafficking [46]. High levels of glucose generate hypertonic conditions and block the internalization of the membrane, disrupting clathrin assembly and recycling during CME [47]. Studies have reported that a major cellular internalization of anionic nanoparticles occurs via pinocytosis, macropinocytosis, and CME [48,49]. In our study, we observed that the uptake of IC occurs predominantly via pinocytosis, and CME is preferred as a minor pathway (Figure 5J–L. The zeta potential analysis of IC indicated a strongly-negative surface charge (Figure S1b). The charged nanoparticles exhibited superior cell membrane adhesion and penetration compared to the neutral nanoparticles. The cationic nanoparticles show higher cytotoxicity due to their stronger interaction with the cell membrane compared to anionic particles [50]. Hence, the cytocompatibility of IC observed in our study (6.25 µg/mL, 12.5 µg/mL, and 25 µg/mL) (Figure 4A) could be attributed to their strong anionic surface charge. In addition, the hydrophilic nature of nanoparticles enhances the wrapping of cell membranes around them for internalization [51]. Therefore, we postulate that the strong anionic charge and hydrophilic nature of IC trigger rapid and efficient cellular uptake.

The denaturation of proteins from damaged cells during inflammatory events, such as arthritis, is commonly observed [34]. Indirectly inhibiting the denaturation of proteins indicates the anti-inflammatory potential of the drug [33]. In our study, we observed that IC could potentially inhibit the denaturation of protein similar to the anti-inflammatory compound 5ASA. IC exhibited the concentration-dependent protection of protein from denaturation. The anti-inflammatory potential of IC could potentially be the result of the bioactive elements present in AC [3,4,6]. The anti-inflammatory action of AC in C2C12 myoblasts has been reported for the first time in our study. Although the predominant source of IL-6 is lymphocytes and macrophages, the potential of skeletal muscle cells to synthesize IL-6 has been well reported since 2003 [52,53,54,55,56]. The potential of skeletal myoblasts and myotubes to enhance IL-6 protein and mRNA expression levels in response to stimuli such as LPS, peptidoglycan, serum amyloid A, pyrogallol, H_2_O_2,_ ROS, and xanthine/xanthine oxidase via TLR2/TLR4 signaling has been reported [47,48,49,50]. Frost et al. reported a 6–8-fold enhancement in IL-6 protein levels and a 5–10-fold increment in IL-6 mRNA levels in C2C12 myoblasts stimulated with LPS [52]. In our study, we show that the LPS (100 ng/mL) stimulation of C2C12 myoblasts and myotubes results in >100 pg/mL IL-6 protein expression in the culture supernatant. Here, we demonstrated that the IL-6 inhibitory action of IC in LPS-stimulated C2C12 myoblast cells is comparable to that of AC. Hence, we prove here that IC is potentially capable of restricting the host inflammatory immune responses, and the complexation of AC extracts with BCD did not alter their anti-inflammatory action in C2C12 myoblasts. Further molecular studies are required to gain a deeper understanding of the IC-stimulated inhibition of the IL-6 signaling pathway.

The inhibition of tumor cell migration, invasion, and proliferation are some of the ways by which AC demonstrates anti-cancer properties [1,2,3,57]. Since the migration of skeletal myoblast cells is a critical event in successful muscle regeneration, we were curious to understand the effect of AC in C2C12 myoblast migration and proliferation. The inhibitory effect of AC in cell migration was evident in C2C12 myoblasts, as observed from the in vitro wound-healing assay (Figure 7C). However, we noticed that IC could potentially improve C2C12 myoblast migration and proliferation (Figure 4B). Our results indicated that the inclusion of AC within the BCD cavity improved its stability, bioavailability, and bioactivity, thus, enabling the improved migration and proliferation of C2C12 myoblast cells. To gain a molecular-level understanding of the action of IC, mRNA and protein expression studies were performed. lncRNAs play critical roles in the proliferation, migration, and differentiation of skeletal muscle cells during development [58]. lncRNA NEAT-1, MEG-3, and SYISL are the three major lncRNAs reported to be involved in regulating the migration and proliferation of skeletal muscle progenitor cells [58,59,60,61]. Hence, we performed RT-PCR studies to understand the influence of IC on the mRNA expression of these lncRNAs. In our study, we noted that the LPS-induced suppression of lncRNA NEAT-1 and SYISL expression was potentially reversed by IC (25 µg/mL) (Figure 7E,F). Dai et al. also reported that LPS downregulated the expression of lncRNA NEAT-1 in MG63 cells [61].

The DNA damage response mechanism involving p53 is majorly involved in the downregulation of lncRNA NEAT-1 [61]. However, whether p53-mediated events are responsible for the LPS-induced downregulation of lncRNA NEAT-1 and the influence of IC in reversing this effect needs further investigation. Our study also showed that IC upregulated the expression of lncRNA SYISL. The lncRNA NEAT-1 and SYISL enhanced the proliferation and migration of C2C12 myoblasts and played a crucial role in muscle regeneration [58,59]. To enhance proliferation, they guide and promote the binding potential of EZH2 (enhancer of zeste homolog 2) at the promoters of the p21 gene, a cyclin-dependent kinase inhibitor, to prevent its expression [58]. lncRNA SYISL also silenced the expression of myosin heavy chain (Myh4), muscle creatin kinase (MCK), and myogenin (MyoG) in a similar manner [57]. Therefore, we suggest that IC could potentially enhance the EZH2/PRC2-mediated inhibition of p21 via lncRNA NEAT-1 and lncRNA SYSIL to promote the proliferation of C2C12 myoblasts.

lncRNA NEAT-1 can promote cell transition from the epithelial to the mesenchymal state by enhancing the expression of MMP-9, MMP-2, and N-cadherin and reducing E-cadherin expression [61,62]. In addition, Zhang et al. observed that lncRNA NEAT-1 can promote colorectal cancer metastasis by activating the DDX5/β-catenin/Wnt pathway [63]. DDX5 is known to protect β-catenin in the cytoplasm from degradation. In the nucleus, DDX5 binds to TCF4 and β-catenin directly and activates the transcription of β-catenin [63,64]. In this study, we observed that IC could promote the protein expression of β-catenin and the EMT marker, N-cadherin. Since lncRNA NEAT-1 is concentrated both in the nucleus and cytoplasm, we postulate that IC promoted the migration of C2C12 myoblasts via lncRNA NEAT-1 in three ways. First, in the nucleus, by activating the lncRNA NEAT-1/DDX5/β-catenin pathway and positively influencing β-catenin transcriptional activity. Second, in the cytoplasm, lncRNA NEAT-1 stabilizes DDX5 post-translationally and makes it available for protecting β-catenin from degradation, thereby initiating the β-catenin/Wnt pathway. Third, by promoting the protein expression of N-cadherin. In our study, we noticed that IC potentially enhanced the protein expression of the pPKC α/β isoform. The activation of PKC directly initiates the β-catenin-mediated signaling pathway, resulting in cell motility [65]. The activated PKC isoforms have been reported to be critical for Wnt5a stability and signaling events [66]. Therefore, we speculate that IC promoted C2C12 myoblast migration by activating the lncRNAs and the downstream effector proteins of the Wnt5a-involved signaling pathway.

## 5. Conclusions

In conclusion, the potential of AC can be employed for effective skeletal myogenesis by modifying it using BCD. The formulated hydrophilic IC exhibited anti-inflammatory properties and an anionic surface charge and were internalized via pinocytosis. It enhanced the migration and proliferation of C2C12 myoblasts via promoting lncRNA NEAT-1, SYSIL, and pPKC/β-catenin signaling events. The usage of nanocarriers also ensures reduced side effects due to low drug requirements. Our study provides sound fundamental evidence that nanocarrier-modified AC can be employed as a therapeutic strategy for successful muscle regeneration. The present study is a proof of concept demonstrated using murine C2C12 myoblast cells. Further studies on the fate of IC after pinocytosis, their release from pinocytic vesicles, and how they induce the expression of lncRNAs assisting skeletal myoblasts migration are essential to understand the complete therapeutic profile of IC.

## Data Availability

All data are presented within this article and Supplementary Material.

## Supplementary Materials

The following supporting information can be downloaded at: https://www.mdpi.com/article/10.3390/cells11162512/s1, Section S1: Nano characterization studies on IC. Figure S1: DLS and zeta potential analysis of IC; Figure S2: Fluorescent microscopic analysis of C2C12 myoblasts treated with (a) AO, (b) IC and (c) AO-IC. Fluorescent spectrophotometric analysis of (d) IC, AO-IC and (e) AO; Section S2. IC improves C2C12 myoblasts migration. Figure S3: Comparison of C2C12 myoblasts migration after treatment with 25 μg/mL of IC and AC at 0 h and 24. (a) cell migration percentage and (b) in vitro wound healing analysis. Section S3. Western blot Raw files. Figure S4. Western blot raw files of (a) N-cadherin, (b) N-cadherin-β-actin, (c) β-catenin, (d) β-catenin-β-actin, (e) p-PKC α/β II, (f) PKC α/β II-β-actin. [27,28,30,37,38,41,44].
