# Supplementary material for: Nano Modification of Antrodia Cinnamomea Exhibits Anti-Inflammatory Action and Improves the Migratory Potential of Myogenic Progenitors
Source: Cells. 2022 Aug 12;11(16):2512. doi: 10.3390/cells11162512 (PMC9406806; doi:10.3390/cells11162512)
Supplement: Supplementary file 1 [file cells-11-02512-s001.zip › cells-1829845-supplementary.pdf]

Supplementary Material

1. Nano characterization studies on IC

1.1. Zeta potential and DLS analysis

The zeta potential and DLS analysis indicated that IC exhibited a hydrodynamic size of 252.6±3.2 nm with a dispersity index of 0.340 ± 0.05 and a strongly negative surface charge of -37.24 ± 1.55 (Figure S1 (a)-(b)). The low dispersity index of IC indicated uniformity of the particles with a lower tendency to aggregate. The anionic nature of IC indicated their superior stability and suitability for cellular uptake.

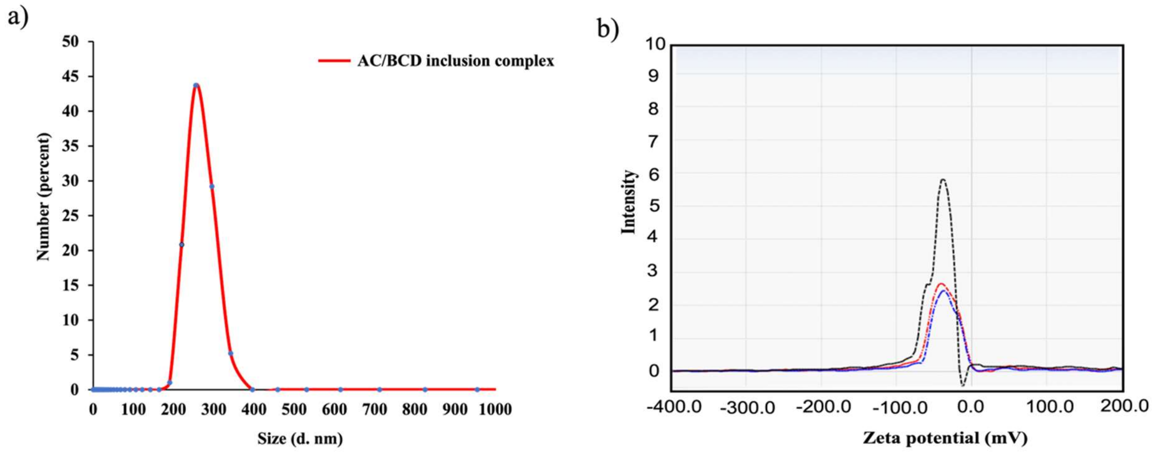

Figure S1: DLS and zeta potential analysis of IC

1.2. SEM Analysis

The SEM analysis demonstrated solid-state microstructure differences between IC, BCD, and AC. The BCD appeared as three-dimensional parallelogram crystals having plate morphology (Figure 1 (d)) whereas the pure drug AC resembled three-dimensional crystals with an irregular shape (Figure 1 (e)). Interestingly, the association of BCD with AC resulted in the complex formation with significant morphological differences. The original morphology of the parent compounds disappeared in IC (Figure 1 (f) and (g)). High magnification imaging of IC showed the presence of spherical beads on the surface of IC (Figure 1(g)). In addition, IC exhibited porous and amorphous nature. The morphological differences present in them indicated successful molecular encapsulation of AC by BCD and the formation of the inclusion complex.

1.3. TGA/DSC analysis

To investigate the thermal stability of BCD, AC, and IC, TGA/DCS analysis was performed. The TGA curve obtained for BCD indicated weight loss at two temperatures (Figure 2 (a) (blue)). The initial weight loss of 10% was observed at 50℃ due to the loss of water molecules present in its cavity and 65% weight loss upon heating at 300℃ due to the decomposition of the macromolecules present in it [1]. AC demonstrated gradual weight loss initiating from 180℃ (Figure 2 (b) (blue)) and a drastic weight loss (75%) at 380℃. About 98% weight loss was observed in AC at 500℃. Interestingly, the weight loss pattern of IC was different from the parent compounds. About 20% weight loss in IC was observed when the complex was heated up to 300℃ (Figure 2 (c) (blue)). The drastic weight loss observed at 348ºC could be attributed to the decomposition of BCD macromolecules present in IC and the percentage of weight loss (50%) indicated that IC demonstrated superior heat stability than parent compounds with only 90% weight loss at 500℃. The DSC analysis indicated that BCD exhibited a wide endothermic peak at 48ºC due to the release of bound water of crystallization from its cavity and the endothermic peak at

225℃ is attributed to its phase transition [2-3](Figure 2 (a) (red)). The peak at 300℃ indicated an exothermic reaction due to crystallization explaining the partial crystalline nature of BCD [4]. AC exhibited an extremely wide endothermic peak at 225 ºC which indicated the endothermic reaction occurring due to the melting of AC (Figure 2 (b) (red)). This was followed by a narrow exothermic peak at 365℃ indicating an exothermic reaction of crystallization explaining the crystalline nature of the AC. Interestingly, IC exhibited a narrow endothermic peak at 48℃ due to the elimination of water molecules in the cavity of BCD upon inclusion of AC (Figure 2 (c) (red)). The absence of an endothermic peak at 225℃ and the presence of a new sharp endo peak at 320℃ indicated the formation of an inclusion complex with a higher melting point. Thus, IC exhibited high thermal stability with an amorphous structure.

1.4. FT-IR analysis

FT-IR spectroscopy was performed to understand the formation of IC between AC and BCD by analyzing the changes in infra-red (IR) spectral peak position, intensity, and shape. The spectrum obtained for BCD (Figure 2 (e)) exhibited characteristic peaks at 3309 cm^{-1} due to O-H stretching vibrations resulting from the intermolecular hydrogen bonds present in them [5]. The sharp transmittance observed at 2925 cm^{-1} is attributed to the C-H stretching vibration [6-7]. The absorption peak present at 1643 cm^{-1} is due to H-O-H bending. The peak at 1153 cm^{-1} corresponds to the asymmetric vibrations of C-O-C stretching while the transmittance observed at 1020 cm^{-1} is due to symmetric stretching vibrations of C-O-C bonds [3]. The major absorption peaks observed in the IR spectrum of AC (Figure 2 (f)) are at 3351 cm^{-1} pertaining to the O-H stretching vibration, 2927 cm^{-1} corresponding to the C-H stretching vibration, 2358 cm^{-1} due to O=C=O stretching vibration, 1673 cm^{-1} (C-H bending vibration), 1375 cm^{-1} (O-H bending), 1155 cm^{-1} (C-O stretching), 1232 cm^{-1} (C=O stretching), 1025 cm^{-1} (C-O-C stretching vibration) and 89^4 cm^{-1} due to C=C bending. Figure 2 (g) shows merged IR spectra obtained for BCD, AC, and IC. The spectrum obtained for IC coincided with the absorption features observed in BCD (Figure 2 (d)). The signature absorption peaks of AC were completely masked by the intense peaks of BCD in IC. Three major spectral changes were observed in IC after the interaction of AC with BCD. The sharp and intense C-H stretching vibration of AC at 2927 cm^{-1} was shifted to a lower frequency (2921 cm^{-1}) with reduced intensity in IC. The sharp C-H bending vibration at 1673 cm^{-1} was shifted to 1650 cm^{-1} with significant intensity reduction in IC. Similarly, the sharp O-H bending vibration observed at 1375 cm^{-1} was shifted to 1330 cm^{-1} in IC with lower intensity. The C-O stretching at 1155 cm^{-1} in AC slightly intensified in IC at 1153 cm^{-1}. These dominant spectral changes in IC indicate the successful inclusion of AC in the BCD cavity.

1.5. UV-visible spectroscopic analysis and in vitro drug release study

The absorption spectra of BCD, AC, and IC are shown in Figure 3 (a). BCD did not exhibit UV absorption throughout the wavelength tested. AC exhibited a strong absorption maximum at 267 nm in the aqueous solution. Interestingly, the absorption spectra of IC indicated a hyperchromic (blue shift) to 264 nm (about 6 nm shift to the lower wavelength) with reduced absorbance. Thus, we concluded that BCD provides a suitable interactive environment for AC in its hydrophobic cavity to form IC. The in-vitro drug release study indicated that IC exhibited enhanced and sustained drug release in the medium (PBS pH 7.4) compared to AC at respective time points (Figure 3 (b)). The dissolution of AC was observed to be low in the medium due to its hydrophobic nature. The association of BCD enhanced the dissolution profile of the complex and complete drug release was observed within 24 h.

1.6. Fluorescent spectroscopic analysis

The fluorescent spectral changes (Figure 3 (c)) indicated that the AC exhibited a single broad emission peak at 400 nm with low fluorescent intensity. Interestingly, the association of BCD with AC enhanced the fluorescent intensity ten times with a shift to the blue spectral region at 380 nm. We noted that the fluorescent behavior of IC was similar to BCD. A strong surface interaction between BCD and AC greatly enhanced the fluorescent intensity of AC. These results indicated the transfer of AC from a protic environment to an aprotic condition (cavity of BCD) to form IC. For the fluorescent spectral analysis, the excitation and emission wavelength were fixed at 320 nm and 360-500 nm respectively. The fluorescent spectra of AO-IC indicated that the interaction of AO with IC caused a significant blue shift from 502 nm (AO) to 380 nm (AO-IC) (Figure S2 (d)-(e)). AO-IC exhibited fluorescent emission characteristic similar to IC at 380 nm. Interestingly, the association of AO with IC highly intensified (1.5 times) the fluorescence emission property of IC (Figure S2 (d)). To understand further, the C2C12 myoblasts were treated with 25 μg/ml AO, AO-IC and IC for 3 h and analyzed using fluorescent microscope. Our study clearly demonstrated that IC without AO association was unable to impart fluorescent property upon cellular uptake (Figure S2 (b)). Hence, cells treated with IC was observed to be non-fluorescent. Interestingly, the complexation of AO with IC contributed fluorescent property to IC (Figure S2 (c)). In addition, we observed that the C2C12 myoblast cells treated with

AO-IC exhibited superior fluorescent property compared to the cells treated with AO (Figure S2 (a)). From these studies, we concluded that complexation of AO with IC provided high intensity fluorescent property to IC and can be easily taken up by the cells.

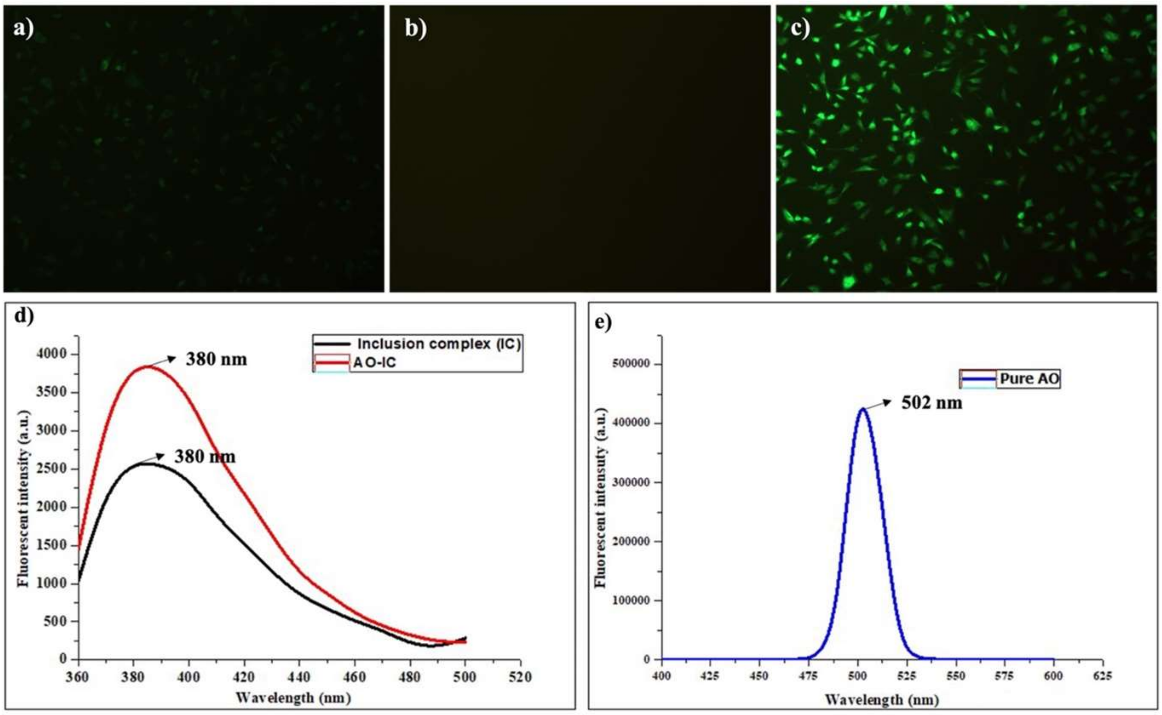

Figure S2. Fluorescent microscopic analysis of C2C12 myoblasts treated with (a) AO, (b) IC and (c) AO-IC. Fluorescent spectrophotometric analysis of (d) IC, AO-IC and (e) AO

2. IC promotes C2C12 myoblasts migration

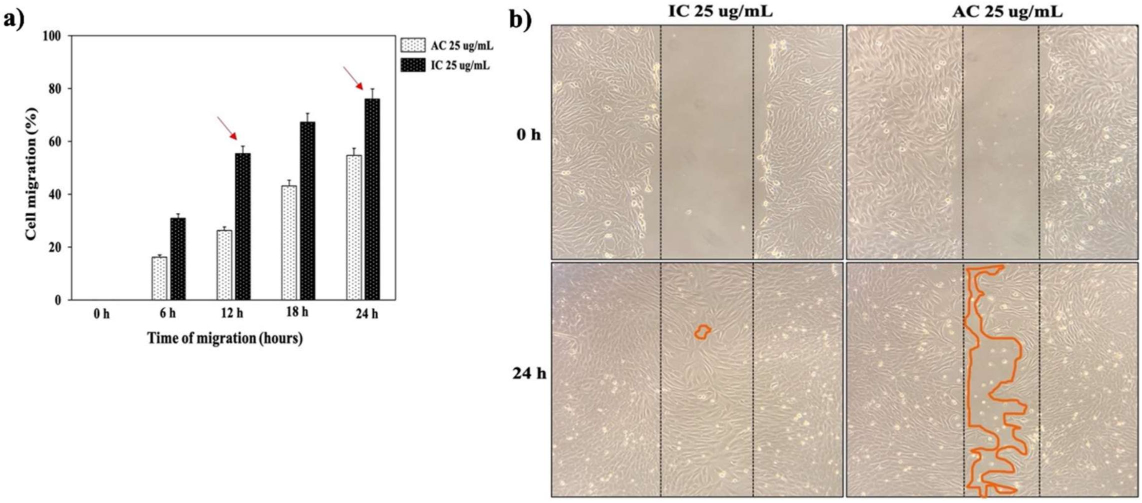

Figure S3**:** Comparison of C2C12 myoblasts migration after treatment with 25 μg/ml of IC and AC at 0h and 24 (a) Cell migration percentage and (b) in vitro wound healing analysis.

3. Western blot Raw files

a)

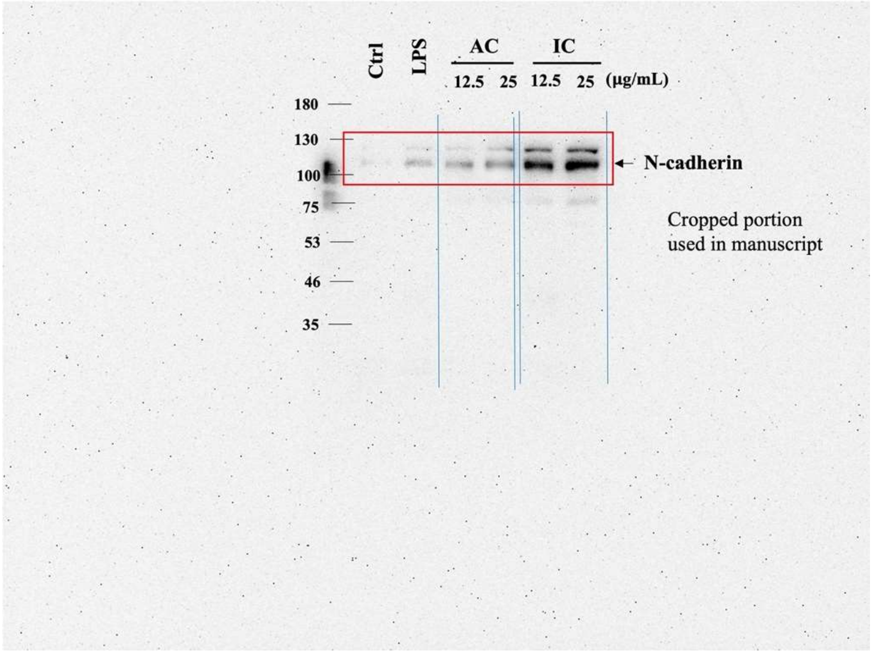

b)

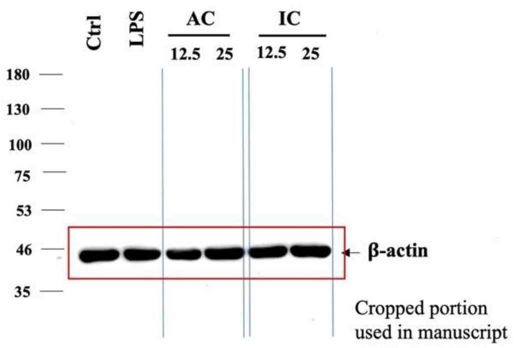

c)

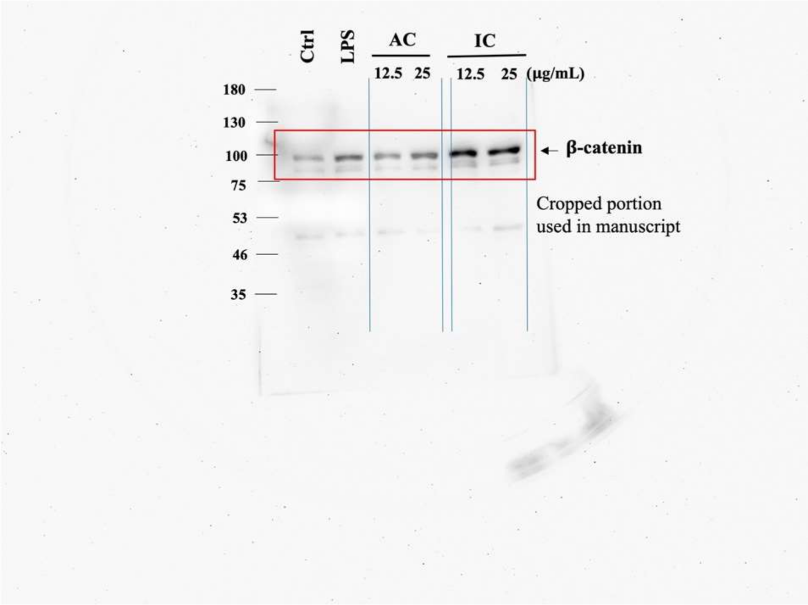
Ctrl
LPS
AC
IC
12.5
25
12.5
25
(µg/mL)
180
130
100
75
53
46
35
β-catenin
Cropped portion used in manuscript

d)

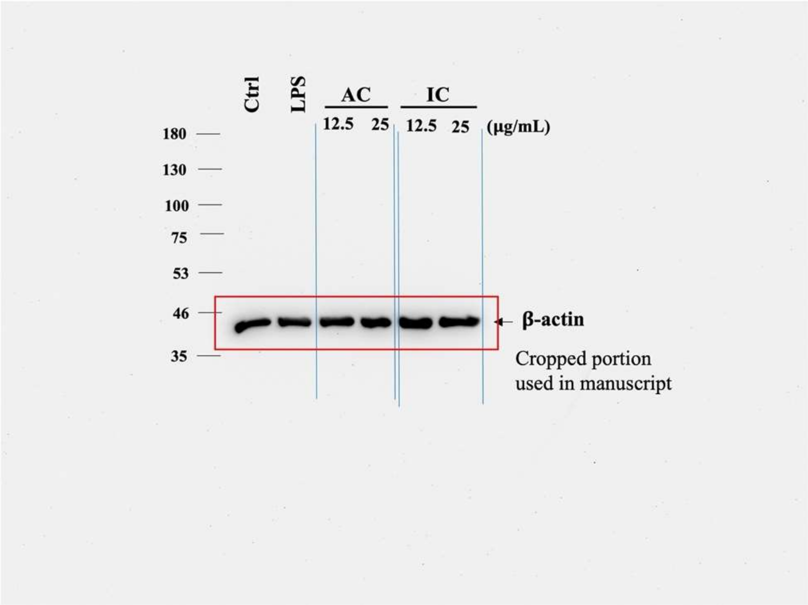
Ctrl
LPS
AC
IC
12.5
25
12.5
25
(µg/mL)
180
130
100
75
53
46
35
β-actin
Cropped portion used in manuscript

e)

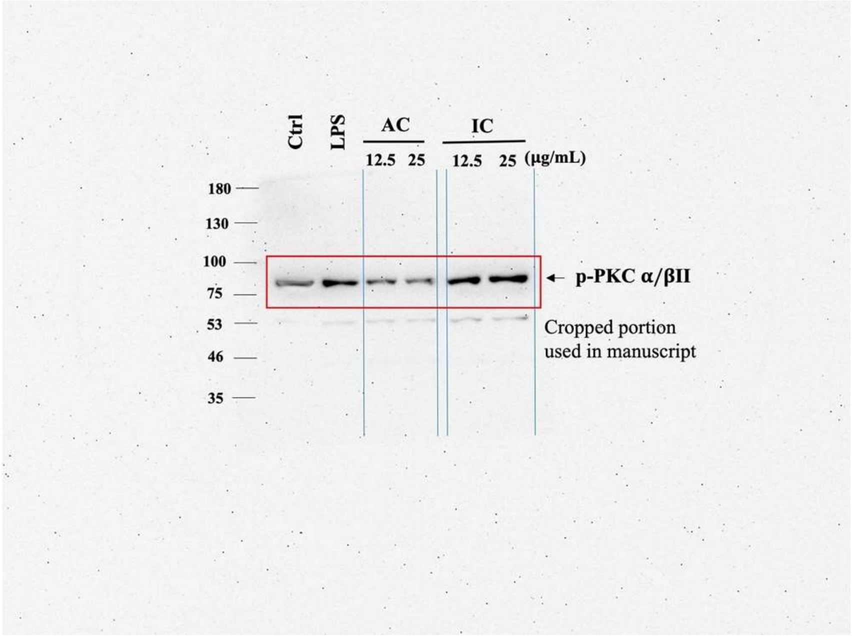

f)

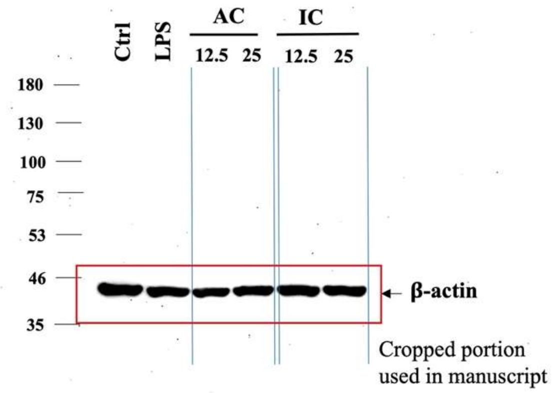

Figure S4. Western blot raw files of (a) N-cadherin, (b) N-cadherin-β-actin, (c) β-catenin, (d) β-catenin-β-actin, (e) p-PKCα/β II, (f) PKCα/β II-β-actin. The exposure time for N-cadherin, β-catenin and p-PKC was 1 minutes 30 seconds. In all the samples, strong β-actin signals were obtained with an exposure time of 30 seconds.

References

1. Sambasevam, K.P.; Mohamad, S.; Sarih, N.M.; Ismail, N.A. Synthesis and characterization of the inclusion complex of β-cyclodextrin and azomethine. *Int J Mol Sci* **2013**, 14, 3671-82. doi: 10.3390/ijms14023671.

2. Abou-Okeil, A.; Rehan, M.; El-Sawy, S.M.; El-Bisi, M.K.; Ahmed-Farid, O.A.; Abdel-Mohdy, F.A. Lidocaine/β-cyclodextrin inclusion complex as drug delivery system. *Eur Polym J* **2018**, 108, 304-10. doi: 10.1016/j.eurpolymj.2018.09.016.N.

3. Morin, N.; Chilouet, A.; Millet, J.; Rouland, J.C. Bifonazole-β-cyclodextrin Inclusion Complexes. Thermal analysis and X-ray powder diffraction study. *J Therm Anal Calorim* **2000**, 62, 187-201. doi:10.1023/A:1010127231416.

4. Louiz, S.; Labiadh, H.; Abderrahim, R. Synthesis and spectroscopy studies of the inclusion complex of 3-amino-5-methyl pyrazole with beta-cyclodextrin. *Spectrochim Acta A Mol Biomol Spectrosc* **2015**, 134, 276-82. doi: 10.1016/j.saa.2014.06.028

5. Rachmawati, H.; Edityaningrum, C.A.; Mauludin, R. Molecular inclusion complex of curcumin–β-cyclodextrin nanoparticle to enhance curcumin skin permeability from hydrophilic matrix gel. *AAPS PharmSciTech* **2013**, 14, 1303-12. doi: 10.1208/s12249-013-0023-5.

6. Al-Marzouqi, A.H.; Shehatta, I.; Jobe, B.; Dowaidar, A. Phase solubility and inclusion complex of itraconazole with β-cyclodextrin using supercritical carbon dioxide. *J Pharm Sci* **2006**, 95, 292-304. doi: 10.1002/jps.20535.

7. Loh, G.O.; Tan, Y.T.; Peh, K.K. Enhancement of norfloxacin solubility via inclusion complexation with β-cyclodextrin and its derivative hydroxypropyl-β-cyclodextrin. *Asian J Pharm Sci* **2016**, 11, 536-46. doi: 10.1016/j.ajps.2016.02.009
